# Update of P2X receptor properties and their pharmacology: IUPHAR Review 30

**DOI:** 10.1111/bph.15299

**Published:** 2020-12-21

**Authors:** Peter Illes, Christa E. Müller, Kenneth A. Jacobson, Thomas Grutter, Annette Nicke, Samuel J. Fountain, Charles Kennedy, Günther Schmalzing, Michael F. Jarvis, Stanko S. Stojilkovic, Brian F. King, Francesco Di Virgilio

**Affiliations:** 1 Rudolf Boehm Institute for Pharmacology and Toxicology, University of Leipzig, Leipzig, Germany; 2 International Collaborative Centre on Big Science Plan for Purinergic Signalling, Chengdu University of Traditional Chinese Medicine, Chengdu, China; 3 PharmaCenter Bonn, Pharmaceutical Institute, Pharmaceutical & Medicinal Chemistry, University of Bonn, Bonn, Germany; 4 Molecular Recognition Section, Laboratory of Bioorganic Chemistry, National Institute of Diabetes and Digestive and Kidney Diseases, National Institutes of Health, Bethesda, MD, USA; 5 University of Strasbourg, Centre National de la Recherche Scientifique, CAMB UMR 7199, Strasbourg, France; 6 Walther Straub Institute for Pharmacology and Toxicology, Ludwig-Maximilians-Universität München, Munich, Germany; 7 Faculty of Science, University of East Anglia, Norwich, UK; 8 Strathclyde Institute of Pharmacy and Biomedical Sciences, University of Strathclyde, Glasgow, UK; 9 Institute of Clinical Pharmacology, RWTH Aachen University, Aachen, Germany; 10 Global Medical Affairs, Abbvie, Inc., North Chicago, IL, USA; 11 Section on Cellular Signaling, The Eunice Kennedy Shiver National Institute of Child Health and Human Development, National Institutes of Health, Bethesda, MD, USA; 12 Department of Neuroscience, Physiology and Pharmacology, University College London, London, UK; 13 Department of Medical Sciences, Section of Experimental Medicine, University of Ferrara, Ferrara, Italy

**Keywords:** (patho)physiological functions, agonists, antagonists, extracellular ATP, knockout mice, ligand-gated cationic channels, P2X receptors

## Abstract

The known seven mammalian receptor subunits (P2X1–7) form cationic channels gated by ATP. Three subunits compose a receptor channel. Each subunit is a polypeptide consisting of two transmembrane regions (TM1 and TM2), intracellular N- and C-termini, and a bulky extracellular loop. Crystallization allowed the identification of the 3D structure and gating cycle of P2X receptors. The agonist-binding pocket is located at the intersection of two neighbouring subunits. In addition to the mammalian P2X receptors, their primitive ligand-gated counterparts with little structural similarity have also been cloned. Selective agonists for P2X receptor subtypes are not available, but medicinal chemistry supplied a range of subtype-selective antagonists, as well as positive and negative allosteric modulators. Knockout mice and selective antagonists helped to identify pathological functions due to defective P2X receptors, such as male infertility (P2X1), hearing loss (P2X2), pain/cough (P2X3), neuropathic pain (P2X4), inflammatory bone loss (P2X5), and faulty immune reactions (P2X7).

## 1 | INTRODUCTION

ATP, originally assumed to be exclusively the universal energy currency of cells, was proposed by Burnstock (1972) in his classic review to be an extracellular, non-adrenergic, non-cholinergic (NANC) neurotransmitter in smooth muscle organs of the gastrointestinal tract (see Verkhratsky, Zimmermann, Abbracchio, Illes, & Di Virgilio, 2020, for a fuller description of the evidence). After the acceptance of “NANC neurotransmission,” and the broadening of this hypothesis to the “co-transmission” idea (co-storage and co-release of ATP with acetylcholine (ACh) or noradrenaline; Burnstock, 1976), purine and pyrimidine nucleotides were then recognized to be extracellular signalling molecules that co-ordinate the function of almost every cell in the animal organisms, including humans (Burnstock & Knight, 2004). Receptors that are stimulated by these nucleotides have been classified into two types (Burnstock & Kennedy, 1985), the ligand-gated cationic channels termed P2X receptors with seven mammalian subtypes: P2X1–7 (Khakh et al., 2001) and the G protein-coupled P2Y receptors with eight mammalian subtypes: P2Y_1,2,4,6,11–14_ (Abbracchio et al., 2006).

For P2Y receptors, an excellent new IUPHAR review is available, describing in detail the pharmacological data available on this class of receptors (Jacobson et al., 2020). However, our knowledge on P2X receptor nomenclature and subunit properties was last summarized in an official IUPHAR review in 2001 (Khakh et al., 2001), with an interim, and non-official updating in 2009 (Jarvis & Khakh, 2009). Hence, the aim of this review is to present a critical update of the pharmacological properties as well as physiological and pathophysiological functions of P2X receptor.

## 2 | MEDICINAL CHEMISTRY OF P2X RECEPTOR LIGANDS

The mammalian P2X receptors form homotrimeric or heterotrimeric channels, each of which harbours three ATP-binding sites. Table 1 summarizes the molecular and general pharmacological properties (agonist and antagonist potencies) of the seven P2X receptor subtypes. For this purpose, we have updated a most helpful table from a review by Jarvis and Khakh (2009) by including more recently discovered receptor ligands and introducing some recent developments (i.e., generation of knockout [KO] animal models).

### 2.1 | P2X receptor agonists

**FIGURE 1:**
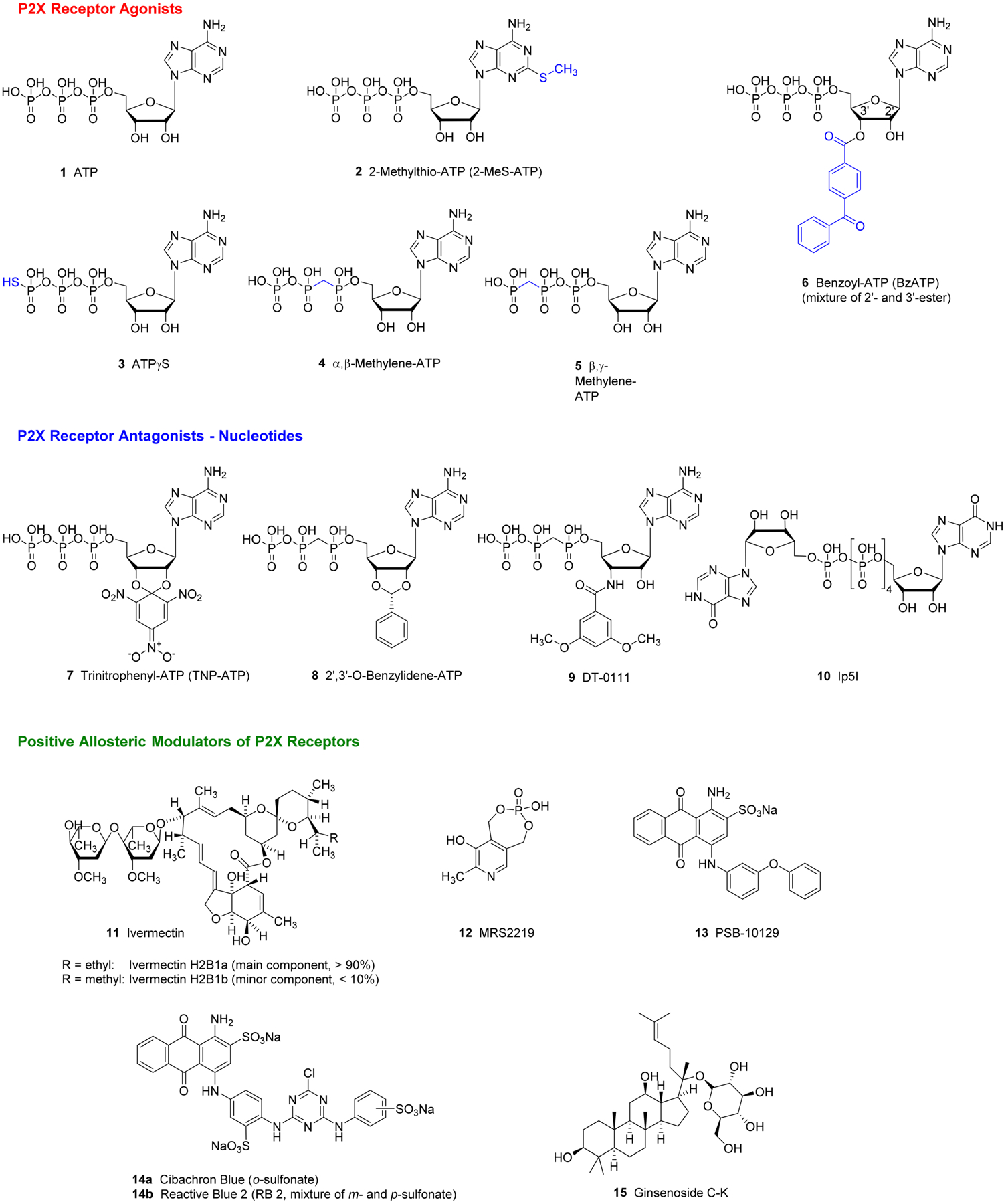
Selected P2X receptor agonists, nucleotide-derived antagonists, and positive allosteric modulators

Despite high conservation of the ATP-binding site in each P2X receptor subtype, there are marked differences in the potency of ATP (**1**) (Table 1). It is active in the low micromolar to submicromolar range at all subtypes, except for the P2X7 receptor, which requires ATP concentrations in the hundreds of micromolar range for activation. P2X receptor agonists structurally derived from ATP have been described (see Figure 1), but agonists with high selectivity for a single subtype are presently not available (Jacobson & Müller, 2016; Lambertucci et al., 2015). 2-Methylthio-ATP (**2**) and ATPγS (**3**) display a profile similar to that of ATP, but both compounds are metabolically more stable. α,β-Methylene ATP (**4**) shows a preference for P2X1 and P2X3 receptors with some-what lower P2X4 receptor potency and much lower P2X7 receptor potency, while β,γ-methylene ATP (**5**) is most potent at P2X1 receptors and has only negligible potency at the other subtypes. 2′ (3′)-*O*-(4-benzoylbenzoyl)ATP (BzATP, **6**) is sometimes described as a selective agonist for P2X7 receptors, but in fact, it has the highest potency at P2X1 receptors followed by P2X3 receptors (for data, see Table 1). Nonetheless, because it is approximately 10 times more potent than ATP, it is frequently used for activation of P2X7 receptors to avoid high, cytotoxic concentrations of ATP. 2′,3′-Substitution with bulky residues, as in trinitrophenyl (TNP)-ATP (**7**) and the P2X3 receptor antagonists **8** and **9** (DT-0111), or dinucleotide formation, as in di-inosine pentaphosphate (Ip_**5**_**I**, **10**), can abolish agonistic activity leading to P2X receptor antagonists.

### 2.2 | Allosteric modulation of P2X receptors by physiological ions, lipids, steroids, and ethanol

P2X receptor function can be allosterically modulated by ions (e.g., Mg^2+^, Ca^2+^, and Zn^2+^; Table 1), steroids, bile acids, and lipids, for example, phosphatidylinositol polyphosphates such as PI(4,5)P_**2**_ (Bernier et al., 2008). The PIPs bind to positively charged amino acids in the cytosolic C-terminal domain and inhibit P2X receptor-mediated currents. The P2X4 receptors, and to some extent the P2X2 receptors, are inhibited by high ethanol concentrations (~100 mM) (Ilyaskin, Sure, Nesterov, Haerteis, & Korbmacher, 2019; Müller, 2015; Sivcev et al., 2019).

### 2.3 | Positive allosteric modulators of P2X receptors

**Ivermectin** (**11**, Figure 1), a CNS penetrant macrocyclic lactone used in veterinary and human medicine as an anti-parasitic agent, interacts with a variety of ion channels (Müller, 2015; Zemkova, Tvrdonova, Bhattacharya, & Jindrichova, 2014) and acts as a positive allosteric modulator (PAM) at P2X4 receptors, facilitating the opening and retarding the closing of the channel in the 100 nM to 3 μM range (Khakh, Proctor, Dunwiddie, Labarca, & Lester, 1999; Priel & Silberberg, 2004). At a similar concentration (3 μM), ivermectin is also active at the human (h), but not rat (r) and mouse (m) P2X7 receptors (Nörenberg et al., 2012). Structural modification of several antagonists has produced derivatives with positive modulatory activity. MRS2219 (**12**) selectively potentiates ATP-induced responses at recombinant rP2X1 receptors expressed in *Xenopus laevis* oocytes, with an EC_50_ of 5.9 μM (Jacobson et al., 1998). Among a series of P2X2 receptor antagonists with an anthraquinone core structure, several derivatives showed positive allosteric modulation. PSB-10129 (**13**) was one of the most potent PAMs of P2X2 receptors (EC_50_ = 489 nM), causing a threefold increase in the maximal ATP-elicited current (Baqi et al., 2011). The anthraquinone derivative Cibacron Blue (**14a**), which is one of the isomers present in Reactive Blue 2 (**14b**), is a PAM of hP2X3 and rP2X4 receptors (Baqi et al., 2011). It is non-selective and may also interact with other P2X receptor subtypes. Recently, ginsenosides, for example, **15**, structurally related to steroids and representing the main constituents of the medicinal plant, ginseng (*Panax ginseng*), were found to act as P2X4 receptor PAMs (Dhuna et al., 2019). The development of PAMs may be a promising approach for drug development, since selectivity for orthosteric agonists will be difficult to achieve.

### 2.4 | Non-selective P2X receptor antagonists

**FIGURE 2:**
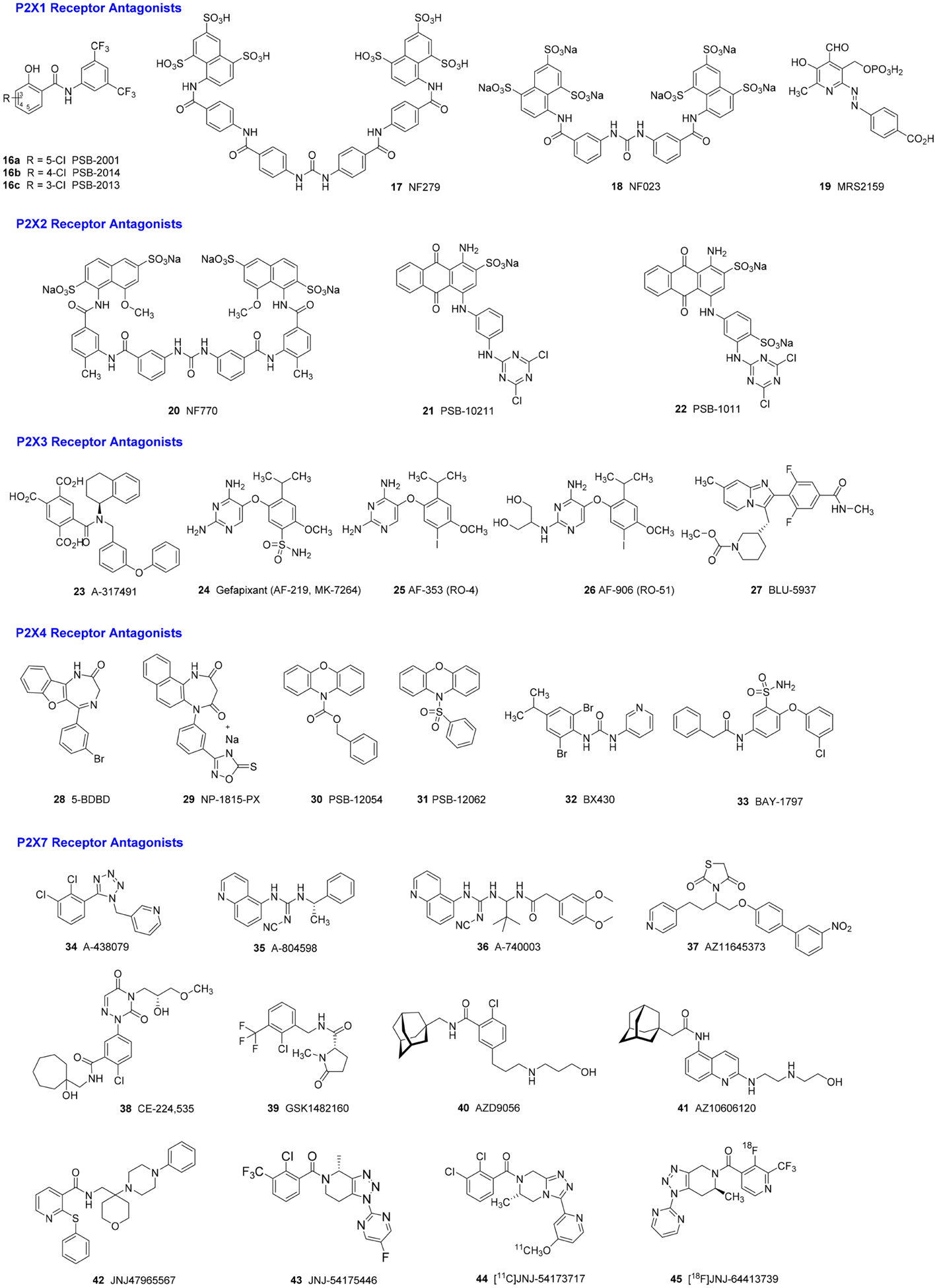
P2X receptor subtype-selective antagonists

Moderately potent, non-selective P2X receptor antagonists include suramin, Reactive Blue 2 (**14b**, Figure 2), PPADS, and iso-PPADS. They are of limited use and should be replaced by more potent and selective antagonists that are now available. The ATP derivative TNP-ATP (**7**) is very potent at P2X1 and P2X3 receptors (low nanomolar IC_50_ values), much less potent at P2X2 and P2X4 receptors, and virtually inactive at P2X7 receptors (Dal Ben et al., 2019). Ip_5_I (**10**) is most potent as a P2X1 receptor antagonist and also blocks P2X3 receptors, but not the heteromeric P2X2/3 receptors, while it potentiates P2X4 receptors (Lambertucci et al., 2015). Recently, aurintricarboxylic acid, a potent inhibitor of nucleases, was reported to strongly block P2X1 and P2X3 receptors in a non-competitive manner (Obrecht et al., 2019).

### 2.5 | P2X1 receptor antagonists

Only a few P2X1 receptor-selective antagonists have been developed (Figure 2). Salicylamide derivatives with high potency and selectivity were recently described (Tian et al., 2020), representing small, uncharged molecules, which act as negative allosteric modulators (NAMs). Some of them, for example, PSB-2014 (**16b**), only partly inhibited hP2X1 receptors. Suramin derivatives, for example, NF279 (**17**) and NF023 (**18**), appear to be competitive antagonists. MRS2159 (**19**), derived from PPADS, binds covalently to the orthosteric binding site of the receptor (Erhardt, Pillarisetti, & Toomey, 2003) but may also block P2X7 receptors (Donnelly-Roberts, Namovic, Han, & Jarvis, 2009).

### 2.6 | P2X2 receptor antagonists

Only a few selective P2X2 receptor antagonists have been developed (Figure 2). The anionic standard P2X receptor antagonists - PPADS, Reactive Blue 2 (**14b**), TNP-ATP (**7**), and suramin - are moderately potent, non-selective P2X2 receptor antagonists. The suramin derivative, NF770 (**20**), is more potent and selective for P2X2 than to the other P2X receptors, and evidence was presented for a competitive mechanism of action (Wolf et al., 2011). Potent and selective P2X2 receptor antagonists related to Reactive Blue 2 have been developed, such as PSB-10211 (**21**) and PSB-1011 (**22**) (Baqi et al., 2011).

### 2.7 | P2X3 receptor antagonists

Many potent, selective P2X3 receptor antagonists have been developed (Ford, 2012; Marucci et al., 2019; Müller, 2010) (Figure 2). One of the first was A-317491 (**23**), a tricarboxylate, which binds to the ATP-binding site, acting as a competitive antagonist (Jarvis et al., 2002). It displays low peroral and CNS bioavailability and high plasma–protein binding. 2′,3′-Benzylidene-ATP (**8**) and related ATP derivatives have submicromolar potency and some P2X3 receptor selectivity (Dal Ben et al., 2019). The 3′-benzamido-ATP derivative, DT-0111 (**9**), was developed as a water-soluble P2X2/3 receptor antagonist suitable for administration by inhalation (Pelleg, Xu, Zhuang, Undem, & Burnstock, 2019). It has an IC_50_ value of 0.3 μM, as well as high selectivity, and represents a drug candidate for chronic obstructive pulmonary disease, chronic cough and overactive urinary bladder (Pelleg, Xu, Zhuang, Undem, & Burnstock, 2019).

A series of allosteric antagonists were discovered in a high-throughput screening campaign centred on the drug trimethoprim (for reviews, see Müller, 2010, 2015). Gefapixant (**24**), named after Geoffrey Burnstock (“Gef” sounds the same as “Geoff”), has been advanced to clinical trials for chronic cough (Morice et al., 2019). Further potent and selective derivatives of this series include AF-353 (**25**) and AF-906 (**26**). AF-353 displays IC_50_ values of 6 nM (hP2X3 receptors), 13 nM (rP2X3 receptors), and 25 nM (hP2X2/3 receptors) and is selective versus P2X1, P2X2, P2X5, and P2X7 receptors (IC_50_ > 10 μM) as well as a range of other targets. It shows peroral bioavailability and is brain penetrant. AF-906 displayed superior pharmacokinetic properties and IC_50_ values of 2 nM (hP2X3 receptors) and 5 nM (hP2X2/3 receptors). The imidazopyridine derivative BLU-5937 (**27**) was reported to be selective for the homomeric P2X3 receptors versus the heteromeric P2X2/3 receptors and therefore, in contrast to gefapixant, is expected not to affect taste sensing. The compound is being evaluated clinically for chronic cough and pruritus (Garceau & Chauret, 2019).

### 2.8 | P2X4 receptor antagonists

The benzodiazepine derivative 5-BDBD (**28**, Figure 2) is a moderately potent (IC_50_ = 0.5 μM), selective allosteric P2X4 receptor antagonist (Abdelrahman et al., 2017). It displays low water solubility and is therefore not easily handled. NP-1815-PX (**29**) is a related compound with increased water solubility (Matsumura et al., 2016). A possibly related compound, NC-2600 (structure not disclosed), entered clinical trials for neuropathic pain, but after a phase I study, no further development has been reported. The allosteric P2X4 receptor antagonist, *N*-(benzyloxycarbonyl)phenoxazine (PSB-12054, **30**), exhibited an IC_50_ of 0.2 μM at the hP2X4, but was less potent at rP2X4 (2.1 μM) and mP2X4 receptors (1.8 μM) (Hernandez-Olmos et al., 2012). A drawback is its high lipophilicity and moderate water solubility. A more water-soluble analogue is PSB-12062 (**31**), the *N*-(*p*-methylphenylsulfonyl)-substituted phenoxazine. It was similarly potent at hP2X4 (IC_50_ = 1.4 μM), rP2X4 (0.9 μM), and mP2X4 receptors (1.8 μM) and showed selectivity versus P2X1, P2X3 and P2X7 receptors (Hernandez-Olmos et al., 2012). The urea derivative **BX430** (**32**) was identified by compound library screening as an allosteric antagonist with an IC_50_ value of 0.5 μM at the hP2X4 receptor. It showed selectivity versus other P2X receptor subtypes but had no effect on mP2X4 and rP2X4 receptors (Ase, Honson, Zaghdane, Pfeifer, & Seguela, 2015). The sulfonamide BAY-1797 (**33**) was recently presented as a compound with an IC_50_ of 210 nM at the hP2X4 receptor and high selectivity (Werner et al., 2019). This antagonist shows high water solubility and no brain penetration. The potent and selective P2X4 receptor antagonist PSB-15417 (structure undisclosed), a brain-penetrant compound, showed high activity in animal models of neuropathic pain (Teixeira et al., 2019).

### 2.9 | P2X7 receptor antagonists

The P2X7 receptor has been the most extensively investigated subtype for drug development, and numerous potent and selective, mainly allosteric, antagonists have been reported (Gelin, Bhattacharya, & Letavic, 2020). The sulfonate dye **Brilliant Blue G** blocks P2X7 receptors and has been used in a number of studies due to its low cost. However, improved P2X7 receptor antagonists with suitable pharmacokinetic properties are available (Gelin, Bhattacharya, & Letavic, 2020; Rech, Bhattacharya, Letavic, & Savall, 2016) and should be used instead. As for many allosteric modulators, species differences have been observed for some P2X7 receptor antagonists. The very first clinical studies were performed with allosteric P2X7 receptor antagonists, such as AZD9056 (**40**) to treat rheumatoid arthritis, but their effectiveness for this indication was not convincing. Useful P2X7 receptor antagonists (**34**–**45**, Figure 2) belong to a range of chemical compound classes. Some, for example, **43**, show high brain permeability. JNJ54175446 (**43**) has been clinically evaluated for the treatment of major depression and bipolar disorders. ^11^C and ^18^F tracers (**44** and **45**) for PET have been developed.

## 3 | GENERAL STRUCTURE OF P2X RECEPTORS

**FIGURE 3:**
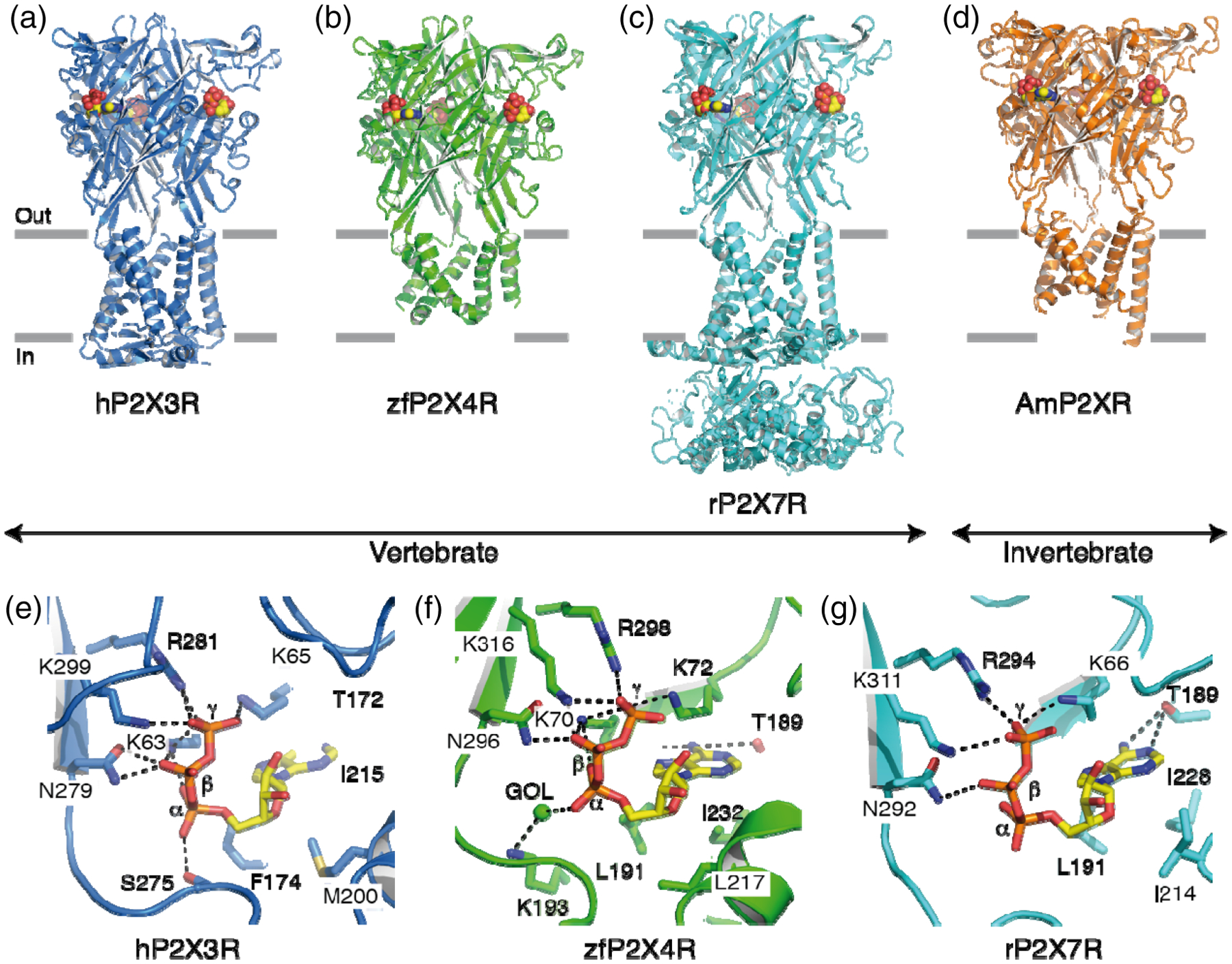
Structures of selected P2X receptors. (a) Structure of the hP2X3 receptor bound to ATP (PBD ID: 5SVK) (Mansoor et al., 2016). The hP2X3 receptor is shown in blue cartoon representation, and ATP is shown as spheres (carbon is yellow, oxygen is red, nitrogen is blue, and phosphorus is orange). Horizontal grey bars indicate the approximate location of the membrane bilayer defining the extracellular (out) and intracellular (in) milieu. (b) Structure of zfP2X4 receptor bound to ATP (4DW1) (Hattori & Gouaux, 2012). The zfP2X4 receptor is shown in green cartoon representation, and ATP is shown as spheres. (c) Structure of the rP2X7 receptor bound to ATP (6U9W) (McCarthy, Yoshioka, & Mansoor, 2019). The rP2X7 receptor is shown in cyan cartoon representation, and ATP is shown as spheres. (d) Structure of the invertebrate AmP2X receptor bound to ATP (5F1C) (Kasuya et al., 2016). The AmP2X receptor is shown in orange cartoon representation, and ATP is shown as spheres. Note the structural similarity between vertebrate and invertebrate P2X receptors. For structures having undergone heavy truncations, membrane spanning helices are lacking in their intracellular sides. (e–g) Close-up views of ATP-binding sites from hP2X3 receptors (e), zfP2X4 receptors (f), and rP2X7 receptors (g). For comparison, views are taken from similar angles, and displayed residues are equivalent across P2X receptors, except for S275 and K193. For those not directly contributing to ATP binding (distance >3.5 Å), equivalent residues are not displayed (e.g., K64 in rP2X7 receptors). ATP is shown in stick representation (carbon is yellow, oxygen red, nitrogen blue, and phosphorus orange) with positions of α-, β-, and γ-phosphate. The oxygen atom from a glycerol molecule (GOL) is shown in sphere representation. Black dashed lines indicate hydrogen bonding (<3.5 Å). AmP2XR, Gulf Coast tick *Amblyomma maculatum* P2X receptor; hP2X3R, human P2X3 receptor; rP2X7R, rat P2X7 receptor; zfP2X4R, zebrafish P2X4 receptor

To date, 27 high-resolution P2X receptor structures have been solved, all within the last decade and all obtained from truncated subunits, except for rP2X7, for which the full-length structure has been solved. Supported by structural data obtained from vertebrate and invertebrate receptors, there is now strong evidence that P2X receptors share similar tertiary and quaternary architecture, further confirming the hypothesis that all P2X receptors belong to the same structural and evolutionary group (Figure 3a–d).

The structural analysis revealed a chalice-like, trimeric assembly of three subunit monomers, with the extracellular domain protruding 70 Å above the plasma membrane plane, and the transmembrane (TM) domain, which is composed of six TM α-helices (two from each subunit) forming the ionic pore, extending approximately 28 Å into the membrane. The intracellular domain protrudes less than the extracellular domain and contains the “cytoplasmic cap,” a highly intertwined region, rich in β-sheets, which is formed by domain swapping of the N- and C-termini of all three subunits. The intracellular region of the rP2X7 receptor contains two additional domains: the cysteine-rich C-cys anchor and the cytoplasmic ballast, both located at the C-terminal end (McCarthy, Yoshioka, & Mansoor, 2019) (Figure 3c).

### 3.1 | Agonist sites

Three ATP-binding sites are found in the extracellular domain within large, interfacial pockets located ~40 Å from the TM domain. ATP-bound structures reveal that the phosphates of ATP bind to several highly conserved, positively charged (e.g., Lys70, Lys72, Arg298, and Lys316 in zfP2X4R) and polar (Asn296) residues (Figure 3e–g). Structures further showed that ATP unexpectedly adopts an unusual U-shaped conformation, allowing the adenine base of ATP to be deeply buried in the binding pocket and recognized by polar (Lys70 and Thr189) and hydrophobic interactions (Leu191 and Ile232). The ribose ring of ATP is recognized by non-polar residues (Leu217), while the O1 and O2 atoms of the ring are rather solvent accessible. Structures of other bound agonists, such as 2-methylthio-ATP (Mansoor et al., 2016) and CTP (Kasuya, Fujiwara, et al., 2017), reveal similar binding modes and orientations, although subtle differences exist, in particular in the base recognition. Interestingly, the entrance to the binding pocket of the P2X7 receptor (<11-Å orifice) (McCarthy, Yoshioka, & Mansoor, 2019) is much narrower than that of other P2X receptors, for example, the P2X3 receptor (17-Å orifice) (Mansoor et al., 2016). This may reduce drug accessibility to the binding pocket and so contribute to the three orders of magnitude lower potency of ATP at the P2X7 receptor compared with other P2X receptor subtypes. Nonetheless, these structural data strongly suggest that the molecular rules governing agonist recognition are highly conserved across P2X receptors and species.

### 3.2 | Antagonist sites

Molecular rules governing competitive antagonist binding seem less stringent than those of agonists. Structures of bound competitive antagonists, such as TNP-ATP or A-317491, reveal significant differences in both binding modes and orientations, when compared with agonist-bound structures. Although TNP-ATP and A-317491 occupy the orthosteric site, they bind more deeply in the binding cleft than ATP, adopting either a Y shape (Mansoor et al., 2016) or an extended conformation (Kasuya, Yamaura, et al., 2017). Compared with the high structural constrains imposed by agonist binding, conformational flexibility of bound competitive antagonists may explain why they do not produce channel opening.

### 3.3 | Allosteric sites

Structures with several bound non-competitive inhibitors also reveal molecular details of allosteric antagonism in P2X receptors (Coddou, Stojilkovic, & Huidobro-Toro, 2011; Habermacher, Dunning, Chataigneau, & Grutter, 2016), and at least two have been resolved at the level of atomic detail: One is located near the apex of the panda P2X7 receptor (Karasawa & Kawate, 2016) and the other one just beneath the orthosteric binding site in the hP2X3 receptor (Wang et al., 2018). Occupancy of these allosteric sites is thought to prevent mechanical motions involved in channel gating. Other allosteric sites are also present in P2X receptors, including sites in the TM that are regulated by phospholipids (Karasawa, Michalski, Mikhelzon, & Kawate, 2017), but their definitive locations have not yet been confirmed by high-resolution structural studies.

### 3.4 | Gating cycle

Of the 27 high-resolution structures, six have been solved in an apparent open channel conformation, bound to ATP, and three in a desensitized, closed channel state, bound to either ATP or 2-methylthio-ATP. Based on these structures, gating cycle models have been proposed for desensitizing (Mansoor et al., 2016) and non-desensitizing channels (McCarthy, Yoshioka, & Mansoor, 2019). Although these structures were all solved in detergents (i.e., in non-native phospholipid bilayers), they provide important clues on channel gating and desensitization mechanisms. Supported by experimental data (see references cited in Habermacher, Dunning, Chataigneau, & Grutter, 2016), it is thought that ATP binding induces a series of structural changes, from tightening of the binding pocket to the outward expansion of the six TM α-helices, leading to channel opening.

Interestingly, the helical pitch of the three innermost TM2 helices changes from an α- to a 3_10_-helix during channel opening. Due to the presence of the cytoplasmic cap, this helical stretching is energetically compensated (Mansoor et al., 2016), which results in the stabilization of the open channel state. However, it appears that during desensitization, TM2 helices recoil as the cytoplasmic cap dis-assembles. Therefore, the structural stability of the cytoplasmic cap appears to tune the rate and extent of desensitization, whereby fast-desensitizing P2X receptors have a less stable cap, compared with slowly or non-desensitizing receptors, such as P2X7 receptors, which possess a more stable cap (McCarthy, Yoshioka, & Mansoor, 2019).

## 4 | P2X RECEPTOR MOUSE MODELS

**FIGURE 4:**
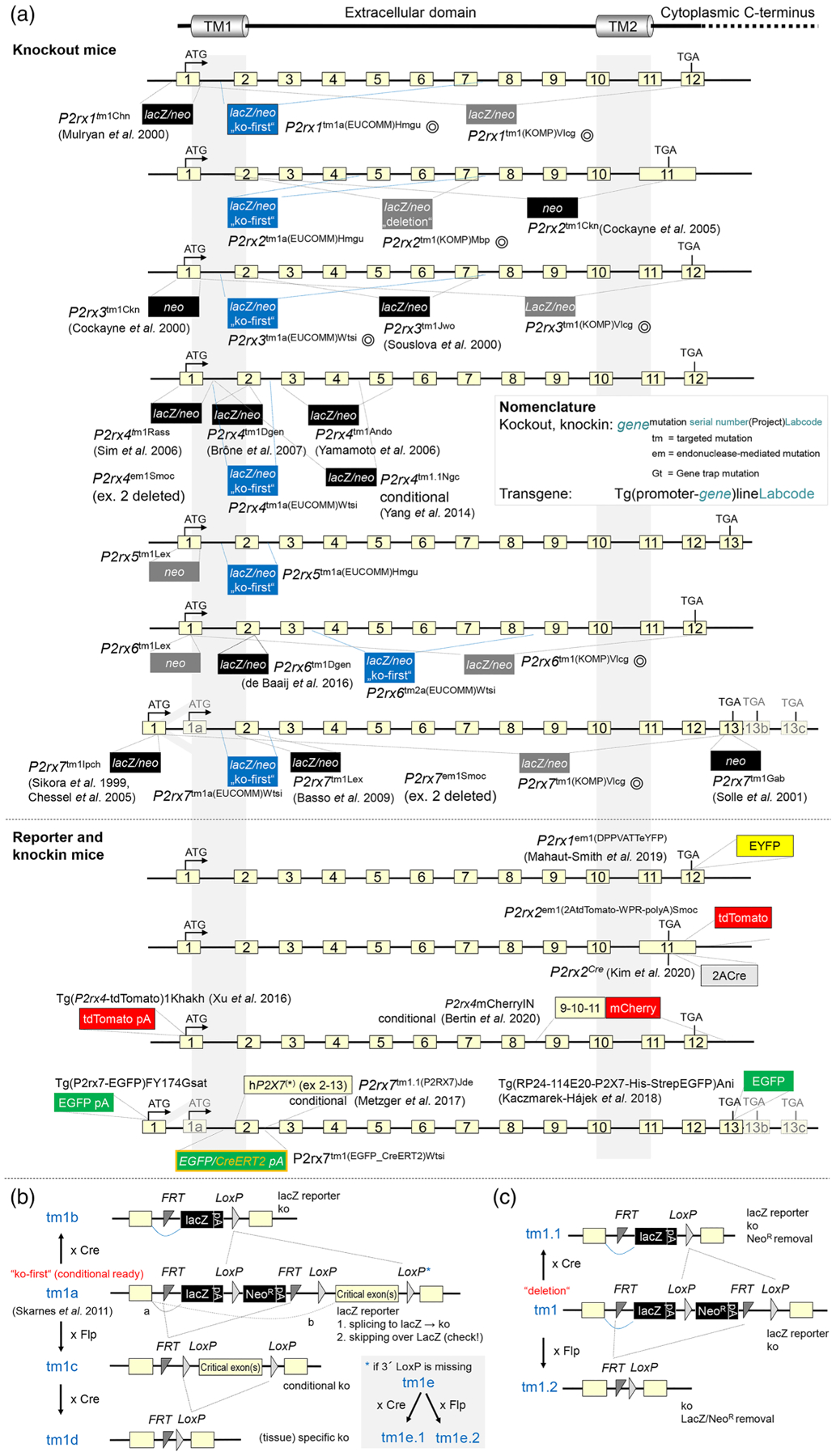
Currently available P2X receptor mouse models according to the literature and Mouse Genome Informatics (MGI)/International Mouse Strain Resource (IMSR). (a) Strategies to target *P2rx1*–*P2rx7* genes for the generation of knockout, knock-in, and transgenic mouse models. The nomenclature according to the current guidelines of the International Committee on Standardized Genetic Nomenclature for Mice is summarized in the inset and found on the mouse nomenclature home page (http://www.informatics.jax.org/mgihome/nomen/index.shtml). P2rx4mCherryIN is not named accordingly, yet. Light yellow boxes represent exons, and black and coloured boxes represent introduced reporter/selection cassettes and/or cDNA. Circles behind the names indicate alleles that are only available in ES cells. In case of conditional strategies, only tm1a alleles (“ko-first”) are shown. These can be further modified as described in (b). Further knockout strains are available from Taconic (deleted exons in brackets) for *P2rx1* (2–7), *P2rx4* (2–4), *P2rx5* (1), *P2rx6* (1–2), and *P2rx7* (2–3) and from TIGM (gene trap vector insertion in brackets) for *P2rx1* (IST14381H9), (IST12457B12), and *P2rx3* (IST10786C2). In addition, *P2rx2*^em1(IMPC)H^, *P2rx4*^Gt(OST340739)Lex^, and Gt (ROSA)26Sor^tm10(RNAi:P2rx7)Rkuhn^ are available. Targeted reporter-tagged insertion with conditional (b) potential (ko-first, conditional ready) and reporter-tagged deletion alleles (c) and the respective nomenclature. Derivative alleles can be obtained through recombinase (Flp or Cre, as indicated)-mediated changes (https://mpi2.github.io/IKMC-knowledgebase/2010/08/24/what-are-the-allele-types.html). The lacZ reporter is supposed to be spliced to the upstream exon 1 (a). However, skipping of lacZ (b) and splicing to the downstream (critical) exon (resulting in functional WT or hypomorph) cannot be excluded and needs to be experimentally determined. The critical exon is supposed to produce a frameshift mutation upon deletion. Note that tm1e represents an unplanned by-product of the original targeting strategy in which the 3′loxP site was lost during recombination but which might still be useful. For detailed information and references, see Kaczmarek-Hajek, Lörinczi, Hausmann, and Nicke (2012), Nicke, Grutter, and Egan (2018), http://www.informatics.jax.org/, and http://www.findmice.org/

The major phenotypes of P2X receptor KO mice are summarized below. For detailed information and references, see Figure 4, Kaczmarek-Hajek, Lörinczi, Hausmann, and Nicke (2012), Jimenez-Mateos, Smith, Nicke and Engel (2019), http://www.informatics.jax.org/, and http://www.findmice.org/. Changes caused by the deletion of individual P2X receptors will be discussed in detail in the specific P2X receptor sections.

Consistent with P2X1 receptor expression, *P2rx1*^*t*m1Chn^ mice show a smooth muscle (90% reduced male fertility and slight hypertension) and a prothrombotic phenotype. Despite abundant P2X2 receptor expression in neuronal and non-neuronal tissues, *P2rx2*^tm1Ckn^ mice present a mild phenotype, with impaired neurotransmission, for example, in pelvic afferent nerves, carotid sinus nerve, and sensory ganglia. A role for cochlear P2X2 receptors in noise adaption and hearing loss was found in *P2rx2*^tm1Ckn^ mice and in humans carrying a P2X2 receptor mutation. *P2rx3*^tm1Ckn^ and *P2rx3*^tm1Jwo^ are less sensitive to inflammatory pain, but not acute noxious thermal (hot plate) and mechanical stimuli. However, using different assays, thermal hyperresponsiveness was observed and compensatory effects were suggested. Like *P2rx2*^tm1Ckn^ mice, *P2rx3*^−/−^ mice showed bladder hyporeflexia and impaired peristalsis and additionally altered hippocampal synaptic plasticity. *P2rx2*/*P2rx3*^Dbl−/−^ mice have gustatory deficits and show developmental abnormalities and high lethality due to pneumonia, probably resulting from reduced ventilatory responses to hypoxia. Surviving mice appear normal. *P2rx4*^*t*m1Rass^ and *P2rx4*^tm1Ando^ mice confirmed the involvement of spinal microglial P2X4 receptors in chronic inflammatory and neuropathic pain. Investigation of *P2rx4*^tm1Ando^ mice also revealed a role of endothelial P2X4 receptors in vascular functions, resulting in high blood pressure (BP). In *P2rx4*^*t*m1Rass^ mice, altered hippocampal synaptic plasticity and perceptual and socio-communicative deficits were described. A mouse model in which P2X4 receptor-mCherry expression in the plasma membrane can be conditionally increased (*P2rx4*mCherryIN) revealed anxiolytic effects and learning deficits. Transgenic hP2X4 receptor-overexpressing mice exhibit increased myocyte contractility, while cardiac-specific P2X4 receptor KO mice (*P2rx4*^tm1.1Ngc^) show more severe heart failure. *P2rx4*^tm1Dgen^ showed abnormal macrophage function. P2X5 receptors are widely distributed in murine tissues; osteoclasts from *P2rx5*^−/−^ mice were shown to have deficits in inflammasome activation and osteoclast maturation under inflammatory conditions (Kim et al., 2017). *P2rx5*^tm1Lex^ mice showed altered immune cell numbers and learning/exploratory behaviour. *P2rx6*^tm1Dgen^ mice showed a pain phenotype.

At least seven *P2rx7*^−/−^ mouse lines have been generated and show deficits in immune function, cytokine release, and reduced inflammatory and neuropathic pain. *P2rx7* deletion is beneficial in numerous pathophysiological and inflammatory conditions. Furthermore, alterations in bone formation (*P2rx7*^tm1Gab^) and an anti-depressant-like profile (*P2rx7*^tm1Lex^) were described. A floxed humanized P2X7 receptor knock-in mouse (*P2rx7*^tm1.1(P2RX7)Jde^) showed an abnormal sleep pattern upon hP2X7R deletion.

Despite invaluable information derived from the early KO models, some caveats need to be considered: *P2rx7*^tm1Ipch^ and *P2rx7*^tm1Gab^ mice express alternative splice variants and incomplete receptor transcripts respectively. The P2X7k variant expressed in the *P2rx7*^tm1Ipch^ mouse is functional and highly expressed in T cells (Kaczmarek-Hajek, Lörinczi, Hausmann, & Nicke, 2012). Also, most published KO mice were generated using embryonic stem (ES) cells derived from 129 mouse strains and the existence and possible functional effects of 129-derived passenger genes or mutations that remain upon back-crossing into other strains need to be considered, as shown for *P2rx4*^*t*m1Rass^ mice (Er-Lukowiak et al., 2020), which contain the “gain-of-function” P2X7Leu451Pro variant. Finally, compensatory effects cannot always be excluded in non-conditional KO mice.

In 2007, the International Knockout Mouse Consortium (IKMC) formed with the aim to knockout each of the >20,000 protein-coding mouse genes in C57BL/6N-derived ES cells. To this end, the KOMP, EUCOMM, NorCOM, and TIGM programs coordinated their strategies to acquire, generate, archive, and distribute KO strains and disseminate the respective data. Within KOMP, two high-throughput gene targeting pipelines were established: (i) a bacterial artificial protein (BAC)-derived targeting vector-based approach (Valenzuela et al., 2003) to create null mutant alleles by preferentially deleting all sequences from the start ATG to the stop codon (used by the VelociGene group) and (ii) a gene-trapping approach (Skarnes et al., 2011) to create “ko-first/conditional-ready” alleles (Figure 4b) for tissue- or time-specific gene deletion (used by Children’s Hospital Oakland Research Institute, Wellcome Trust Sanger Institute, University of California at Davis [KOMP-CSD]). The latter approach is also used in the EU-funded EUCOMM program. In the succeeding EUCOMM Tools program, inducible forms of Cre recombinase (CreERT2) together with an EGFP reporter are now knocked-in into genes with useful expression patterns such as *P2rx7*^tm1(EGFP_CreERT2)Wtsi^.

Furthermore, standardized phenotyping projects were launched, and so far, data for P2X2 (non-significant), P2X4 (clavicle morphology and heart weight), P2X5 (gait and preweaning lethality), P2X6 (cholesterol level and body fat), and P2X7 receptors (cataract and eye morphology) are available at the International Mouse Phenotyping Consortium (IMPC) website (www.mousephenotype.org).

Additionally, P2X reporter mice that express soluble fluorescent reporter proteins or fluorescent protein-tagged receptors are becoming available (Figure 4a). While both the BAC transgenic Tg(*P2rx4*-tdTomato)1Khakh (expressing soluble tdTomato) and the *P2rx4*mCherryIN knock-in mouse (conditionally expressing mCherry-tagged P2X4) reliably reported P2X4 receptor expression (Bertin et al., 2020; Xu et al., 2016), two BAC transgenic P2X7 receptor reporter mice (Tg(P2rx7-EGFP)FY174Gsat and Tg(RP24–114E20-P2X7-His-StrepEGFP)Ani) (expressing soluble and P2X7-fused EGFP, respectively) show highly divergent expression patterns (Kaczmarek-Hajek et al., 2018), further demonstrating the need for careful characterization of mouse models, in particular those generated in high-throughput approaches. Most recently, a knock-in P2X2^Cre^ mouse was described, which upon crossing with Cre-sensitive reporter mice reveals P2X2 receptor expression (Kim et al., 2020).

## 5 | CLONED PRIMITIVE P2X RECEPTORS

The cloning of P2X receptors from primitive organisms including amoeba and algae demonstrates the early utilization of ATP as a fast transmitter molecule (Fountain & Burnstock, 2009). Those cloned to date have low protein sequence homology with mammalian P2X receptors, typically 20–40%, and as such, a different nomenclature has been devised from that of mammalian P2X receptors. This system includes a prefix of an italicized short form of the species the receptor was cloned from, and in cases where multiple subtypes are identified, a letter is denoted in subscript (e.g., *Dd*P2X_A_, *Dictyostelium discoideum* P2X receptor subtype A). The National Centre for Biotechnology (NCBI) accession number is given for each cloned receptor in parenthesis as they are introduced in the following sections. Bioinformatic analysis predicts further primitive P2X receptors (Fountain & Burnstock, 2009), but this section describes those that have been cloned and are reported to form functional P2X receptors.

*D. discoideum* is a soil-living amoeba that transitions to a multicellular developmental life cycle. Five P2X receptors have been cloned, *Dd*P2X_A_ (XM_640286), *Dd*P2X_B_ (XM_638738), *Dd*P2X_C_ (XM_638739), *Dd*P2X_D_ (XM_631676), and *Dd*P2X_E_ (XM_631865). *Dd*P2X_C_ does not form functional channels following heterologous expression in HEK293 cells or *X. laevis* oocytes (Fountain et al., 2007). *Dd*P2X_D_ forms functional channels under low external Na^+^ conditions (Baines et al., 2013). The functional receptors display a rank order in sensitivity to ATP of *Dd*P2X_A_ (EC_50_ 97 μM) >*Dd*P2X_B_ (EC_50_ 266 μM) >*Dd*P2X_E_ (EC_50_ 511 μM). For *Dd*P2X_A_, β,γ-imido-ATP (EC_50_ 15 μM) and α,β-methylene ATP (EC_50_ 95 μM) are full agonists and BzATP is a weak partial agonist (25% activation at 3 mM). β,γ-Imido-ATP is a full agonist at *Dd*P2X_B_ receptors (EC_50_ 85 μM) and weak partial agonist at *Dd*P2X_E_ receptors (22% activation at 3 mM). *Dd*P2X_A_, *Dd*P2X_B_, and *Dd*P2X_E_ receptors are insensitive to suramin, PPADS, and TNP-ATP but inhibited by Cu^2+^ (*Dd*P2X_A_, IC_50_ 40 nM; *Dd*P2X_B_, 85% inhibition at 100 nM; *Dd*P2X_D_, 30% inhibition at 100 nM; and *Dd*P2X_E_, 70% inhibition at 100 nM).

Further P2X receptors with variable sensitivities to known P2X receptor ligands have been described in *Schistosoma mansoni*, *Ostreococcus tauri*, *Monosiga brevicollis*, *Hypsibius dujardini*, *Boophilus microplus*, and *Lymnaea stagnalis* (Fountain, 2013).

## 6 | P2X1 RECEPTOR

### 6.1 | *P2XR1* gene and structure

The *P2RX1* gene (ENSG00000108405) is on human chromosome 17p13.3, is 2,662 bp long, has 12 exons, and encodes a protein of 399 amino acids. The *Ensembl Gene* database lists four splice variants and 262 species orthologues. In common with all other P2X receptor subtypes, 102 species orthologues are present in placental mammals and they are also present in birds, reptiles, and fish, but not in *Caenorhabditis elegans*, *Drosophila melanogaster*, and *Saccharomyces cerevisiae*.

The homomeric P2X1 receptor is a rapidly desensitizing, non-selective, cationic channel, with a relatively high permeability to Ca^2+^ (Egan & Khakh, 2004). Recombinant P2X1 subunits also form functional heteromultimers with the P2X2, P2X4, and P2X5 subunits, but this has not as yet been reported in vivo, with the exception of P2X1/5 (see Kennedy, 2015).

### 6.2 | Expression

*P2RX1* mRNA and P2X1 receptor protein are widely expressed throughout the body (Burnstock & Knight, 2004; Kennedy, 2015). P2X1 is the predominant P2X subunit present in most smooth muscle tissues, including vas deferens, arteries, and urinary bladder. Recently, Mahaut Smith, Evans, and Vial (2019) created a P2X1 receptor-eYFP knock-in mouse, enabling receptor expression to be viewed in live cells. Confirming earlier reports, P2X1 receptor-eYFP fluorescence was seen in urinary bladder and arterial smooth muscle cells, platelets, and megakaryocytes but was absent from the CNS. Furthermore, the receptor is highly mobile within the plasma membrane and likely present in lipid rafts, cholesterol-rich microdomains that are involved in receptor signalling and trafficking.

### 6.3 | Neurotransmission

P2X1 receptors mediate the actions of ATP when it is released as an excitatory co-transmitter with noradrenaline from sympathetic, and with ACh from parasympathetic nerves. Initial evidence depended upon P2X1 receptor desensitization by α,β-methylene ATP (α,β-meATP) or inhibition by antagonists, but the clearest evidence is provided by gene KO (see Kennedy, 2015, and references therein). This greatly decreased the amplitude of sympathetic contractions of mouse vas deferens and was associated with a 90% fall in fertility. Simultaneous KO of α_1A_-adrenoceptors caused total infertility. Thus, P2X1 receptors clearly play a crucial role in male reproductive function. Sympathetic, purinergic co-transmission in arteries mediates vasoconstriction, but its contribution to mean arterial BP is unclear.

Parasympathetic nerves mediate contraction of urinary bladder detrusor smooth muscle, causing voiding of urine. In most species, atropine only partly inhibits these contractions and P2X1 receptor KO abolished the remaining response. Interestingly, P2X1 receptor antagonists failed to mimic the effect of P2X1 receptor KO, and it was suggested that ATP may act at both the homomeric P2X1 receptor and at the P2X1/4 receptor heteromer to elicit urinary bladder contraction (Kennedy, Tasker, Gallacher, & Westfall, 2007).

### 6.4 | Thrombosis

P2X1 receptors mediate Ca^2+^ influx in platelets, but P2X1 receptor KO mice do not show spontaneous bleeding or increased bleeding time (Hechler et al., 2003). Fewer KO mice died, however, in an in vivo model of acute obstruction of the lung microcirculation. Furthermore, thrombi formed by localized damage to arterioles were smaller and easily dispersed. Thus, platelet P2X1 receptors may contribute to thrombi formation, particularly in arterioles, which are narrow and associated with a high shear stress.

### 6.5 | Dysfunctional urinary bladder

Atropine abolishes neurogenic contractions of the healthy urinary bladder in humans, but atropine-resistant contractions appear with increasing age and in chronic disorders, such as interstitial cystitis, idiopathic detrusor instability, and overactive bladder syndrome. They are abolished in vitro by prolonged exposure to α,β-meATP, indicating mediation by P2X1 receptors, though this has yet to be been confirmed using P2X1 receptor antagonists (see Kennedy, 2015).

### 6.6 | Inflammation

ATP released by stressed or damaged cells, or in response to inflammatory stimuli, acts via P2X4, P2X7, P2Y1, P2Y2 and P2Y6 receptors as a damage-associated molecular pattern (DAMP) signalling molecule to elicit pro-inflammatory responses in macrophages and neutrophils (Di Virgilio, Sarti, & Coutinho-Silva, 2020). Lecut et al. (2012) reported that P2X1 receptor KO increased mortality due to LPS-induced endotoxemia, but Maitre et al. (2015) saw a decrease, while there was no difference in the mortality rate from septic shock induced by uropathogenic *Escherichia coli* (Greve et al., 2017). The reasons for this variability are unclear but may reflect differences in the serotype of the pathogenic stimulus used.

Platelets also contribute to inflammation by facilitating immune cell recruitment and activation. In a mouse model of colitis, platelet depletion or P2X1 receptor KO caused intestinal bleeding, leading to macrocytic regenerative anaemia, whereas neutrophil depletion reduced blood loss (Wera et al., 2020). Thus, platelet and neutrophil P2X1 receptors may have protective roles in the inflamed intestine.

### 6.7 | *P2RX1* single nucleotide polymorphisms in cancer

The *HIVE Lab* database lists 86 unique *P2RX1* single nucleotide polymorphisms (SNPs) in cells from multiple types of cancer that change the P2X1 receptor protein sequence. Whether these contribute to the development and/or maintenance of cancer is unknown, but many are predicted to be “probably damaging.” A significant reduction in *P2RX1* mRNA expression was identified in several cases, but it is unclear if this is a cause or effect of the cancer.

## 7 | P2X2 RECEPTOR

### 7.1 | The *P2RX2* gene

The first *PRX2* cDNA encoding the rP2X2 subunit protein (UniProt ID P49653.1) was expression cloned from pheochromocytoma cells using *X. laevis* oocytes (North, 2002). The Ensembl database locates the *hP2RX2* gene (ENSG00000187848) on chromosome 12 (between 132,618,776 and 132,622,388 bp) and predicts eight splice variants. The *P2RX2* genomic structure consists of 11 exons and 10 introns and is highly conserved among human and rat.

### 7.2 | Homotrimeric and heterotrimeric P2X2 receptor proteins

P2X2 receptor subunits assemble during their endoplasmic reticulum-bound synthesis into homotrimers (Aschrafi, Sadtler, Niculescu, Rettinger, & Schmalzing, 2004; Nicke et al., 1998). The contacts between the subunits relevant for trimerization are located in the ectodomain, while the TMs support assembly by restricting the folding space (Duckwitz, Hausmann, Aschrafi, & Schmalzing, 2006). P2X2 receptor subunits are co-expressed with other P2X subtypes in many cell types; in recombinant systems, they can co-assemble to functional and stable heterotrimers, such as P2X2/1, P2X2/3, P2X2/5, and P2X2/6 receptors (Hausmann, Kless, & Schmalzing, 2015).

There is no direct X-ray or cryo-EM structure of a P2X2 receptor available, but X-ray templates from the zfP2X4 receptor (see Section 3) enabled building P2X2 receptor homology models, which proved to be reliable. Achievements of these models include (i) understanding P2X2 receptor channel gating; (ii) localization of potency-determining residues of the P2X2 receptor antagonist NF770 (a suramin derivative) by homology docking; (iii) disclosure of lateral fenestrations as ion access pathways to the channel pore; and (iv) identification of ionic coordination of ATP^4−^ into its binding pocket as an opening mechanism to break a salt bridge that stabilizes the closed state (Hausmann, Kless, & Schmalzing, 2015). The subtype-specific signatures of the homotrimeric P2X2 receptor channel are (i) fast activation by external ATP; (ii) virtually no activation by up to 300-μM external α,β-meATP; and (iii) among all P2X receptor subtypes, the most stable steady-state current during prolonged ATP exposure, with slow or no desensitization (North, 2002). The main active form of ATP at the P2X2 receptor is free ionic ATP (ATP^4−^; EC_50_ 2.0 ± 0.7 μM in divalent-free solution); the Mg^2+^-complexed form MgATP^2−^ binds with much lower affinity and is thus largely ineffective in opening the P2X2 receptor (Li, Silberberg, & Swartz, 2013).

### 7.3 | P2X2 receptor expression and physiological functions

*P2RX2* mRNA and P2X2 receptor protein are expressed abundantly throughout the body on neurons and non-neuronal cells (Cockayne et al., 2005). The Human Brain Atlas (http://proteinatlas.org) points to a particularly high *P2RX2*/P2X2 receptor expression in the hippocampal formation. Compared with the widespread expression in the nervous system, the behavioural phenotype in P2X2 receptor KO mice is remarkably inconspicuous in terms of general excitability of the CNS and sensory and motor function (Cockayne et al., 2005). Essential physiological P2X2 receptor functions are their contribution to the sensitivity of the carotid body to hypoxia by stimulating afferent fibres of the sinus nerve (Rong et al., 2003) and their involvement in taste perception in an epithelial-to-neuronal mode of signalling. Exposure of oral taste receptor cells to taste stimuli releases ATP that activates P2X2 and P2X3 receptors, which are co-expressed in the taste buds that innervate the tongue (Cockayne et al., 2005). P2X2/P2X3 receptor double-KO mice are taste blind to all taste stimuli, while responses to touch, temperature, and menthol remain unaffected. However, single KO of either P2X2 or P2X3 receptors only slightly reduces taste responses (Finger et al., 2005). This implies that in addition to the heterotrimeric P2X2/3 receptors, the homotrimeric P2X2 and/or P2X3 receptors must also be involved in taste perception. A plausible explanation is that presynaptic homotrimeric P2X2 receptors are needed to stimulate ATP secretion via an autocrine positive feedback (Huang et al., 2011).

### 7.4 | P2X2 receptors and auditory system

The P2X2 receptor is abundantly expressed in the cochlea, the sensory hair cells of the organ of Corti, the tectorial membrane, Reissner’s membrane, and spiral ganglion neurons. Sustained elevated noise levels release ATP into the cochlear endolymph via connexin hemichannels. This ATP activates P2X2 receptors on epithelial cells lining the endolymphic compartment. The induced inward current reduces the endocochlear potential and, consequentially, hearing sensitivity. In short, ATP is an auditory neurotransmitter that regulates hearing sensitivity via the P2X2 receptor (see Mittal et al., 2016).

The Orphanet database for rare diseases and orphan drugs assigns *P2RX2* to a single disease-causing germline mutation, autosomal dominant deafness DFNA41. A genomic analysis of a DFNA41 family in China revealed a V^60^L mutation in the *hP2RX2* gene (Yan et al., 2013). Heterozygous family members experienced accelerated noise-induced hearing loss to high frequencies in adolescence. Val^60^Leu-hP2X2 subunits assemble as a constitutively active, ATP-insensitive channel (George, Swartz, & Li, 2019). A second mutation most likely causing autosomal dominant deafness, G^353^R-hP2X2 receptor, was detected in an Italian family. Gly^353^Arg-hP2X2 receptors exhibits alterations in sensitivity to ATP, inward rectification, and ion selectivity (George, Swartz, & Li, 2019). Altogether, these results establish an essential role of the P2X2 receptors for the preservation of hearing.

## 8 | P2X3 AND P2X2/3 RECEPTORS

### 8.1 | *P2rx3* and *P2RX3R* genes and the respective homomeric and heteromeric proteins

The ensemble database reports *mP2rx3* (ENSMUSG00000027071), *rP2rx3* (ENSRNOG00000008552), and *hP2RX3* (ENST00000604659) cDNAs encoding full receptor proteins. The *mP2rx3* has two and the *hP2RX3* only one protein-coding splice variant. The rP2X3 receptor protein was cloned from dorsal root ganglia (DRGs), which when expressed in *X. laevis* oocytes, yielded a channel responding to ATP with a rapidly desensitizing current (Chen et al., 1995). Soon after-wards, it was found that the co-expression of rP2X3 with rP2X2 receptors yielded ATP-activated currents that slowly desensitized and resembled those recorded in rat nodose ganglia (Lewis et al., 1995). Apparently, sensory neurons of the DRG possess a mixture of P2X3 and P2X2/3 receptor channels, while nodose ganglia possess only P2X2/3 receptors. Both P2X3 and P2X2/3 receptors respond to the agonist α,β-meATP with the typical inward currents, although P2X2 receptors are insensitive to α,β-meATP. Originally, it was assumed that P2X2/3 receptors consist of an obligatory combination of two P2X3 and one P2X2 subunit (Jiang et al., 2003), but later, an inverse combination of the two types of subunits [(P2X2)_2_/(P2X3)_1_] was shown to be also functional in expression systems for recombinant receptors (Kowalski et al., 2015).

### 8.2 | P2X3 and P2X2/3 receptor distribution and function

In addition to postsynaptic P2X3 and P2X2/3 receptors located at the cell bodies of sensory neurons, presynaptic P2X3 and P2X2/3 receptors have a facilitatory role to enhance glutamatergic neurotransmission from the central terminals of sensory neurons onto the cell bodies of spinal cord afferent neurons (Khakh & North, 2012). The regulation of P2X3 and P2X2/3 receptor expression in pathophysiology is complex. Both up-regulation and down-regulation of individual receptor subunits have been documented in various experimental models (Bernier, Ase, & Seguela, 2018; North & Jarvis, 2013). This variability is likely dependent on specific contextual influences, including neuroanatomical structure and biochemistry. The ability of ATP to evoke ectopic neuronal hypersensitivity may not be solely dependent on intrinsic P2X3 or P2X2/3 receptor activation, since functional interactions with other P2 receptors, ligand-gated ion channels, and multiple signalling pathways have been described (Bernier, Ase, & Seguela, 2018).

Important insights regarding the physiological roles of P2X2, P2X3 and P2X2/3 receptors were gained through the functional analysis of transgenic and transient gene disrupted (i.e., P2X3 anti-sense) mice (North & Jarvis, 2013). Genetic disruption of each of these individual P2X receptors resulting in a complete loss of receptor expression has been shown to reduce sensory nerve function and a concomitant diminution of nociceptive and hyperactive bladder responses (Ford & Undem, 2013; North & Jarvis, 2013). Transgenic mice lacking both P2X2 and P2X3 receptors also show alterations in taste sensitivity to bitter and sweet substances (Finger et al., 2005).

### 8.3 | P2X3 receptor antagonists in clinical concept trials

Studies using non-selective antagonists or agonist-induced down-regulation of P2X3 receptors provided preliminary evidence for the roles of these receptors in sensory systems (Jarvis & Khakh, 2009). Subsequently, in vivo studies using selective antagonists for P2X3 and P2X2/3 receptors generated evidence that blocking these receptors leads to diminished nociceptive sensitivity in a variety of experimental pain models, reduced bladder reflexes and elevated bladder volume thresholds, and reduced airway sensitivity in preclinical cough models (Ford & Undem, 2013; North & Jarvis, 2013).

Gefapixant (AF-219, MK-7264), the potent and reversible non-competitive antagonist of P2X3 receptors is approximately threefold less potent at P2X2/3 receptors (Richards, Gever, Ford, & Fountain, 2019). Gefapixant and closely related diaminopyrimidine class structural analogues inhibit P2X3 receptor-dependent action potentials in afferent neurons innervating peripheral tissues in a variety of nociceptive, urological, and respiratory models (Ford & Undem, 2013; Richards, Gever, Ford, & Fountain, 2019).

Gefapixant is orally bioavailable, peripherally restricted, and has suitable drug-like properties enabling exploration of its therapeutic potential in humans (Ford & Undem, 2013). A consistent tolerability finding for gefapixant-treated patients is a high prevalence of altered taste sensitivity (dysgeusia; Smith et al., 2020). This appears to be dose dependent, indicating a potential for optimization of a dose to maintain anti-tussive efficacy, while minimizing dysgeusia (Smith et al., 2020).

Based on the known physiological roles of P2X3 and P2X2/3 receptors, their expression on taste buds (Finger et al., 2005) and the phenotype of double-KO mice lacking both P2X2 and P2X3 receptors, gefapixant-mediated dysgeusia is likely mediated by block of the heteromeric P2X2/3 receptors (Garceau & Chauret, 2019). First-generation non-nucleoside P2X3 receptor antagonists show little selectivity in blocking homomeric P2X3 receptors and heteromeric P2X2/3 receptors (North & Jarvis, 2013). However, more recently discovered P2X3 receptor antagonists, including the imidazopyridine, BLU-5937, have stereoselective preferential affinity at P2X3 receptors, with several orders of magnitude lower affinity at P2X2/3 receptors (Garceau & Chauret, 2019). BlU5937 is currently in a proof-of-concept trial for chronic cough.

## 9 | P2X4 RECEPTORS

### 9.1 | The *P2RX4* gene, splice variants, and SNPs

The P2X4 receptor was identified as a distinct member of the P2X family of receptors in 1995–1996, when it was cloned from rat whole brain, hippocampus, and superior cervical ganglia cDNA libraries and characterized by heterologous expression. During 1997–2001, human (*hP2RX4*, ENSG00000135124), mouse, chick, and *X. laevis* receptor cDNAs were also cloned and characterized. Subsequently, rabbit, dog, frog, and zebrafish P2X4 receptors were identified (Kaczmarek-Hajek, Lörinczi, Hausmann, & Nicke, 2012). The *hP2RX4* gene is located at 12q24.32, close to the *P2RX7* gene. The crystal structure of the P2X4 receptor was resolved for the zebrafish receptor (Hattori & Gouaux, 2012; Kawate, Michel, Birdsong, & Gouaux, 2009; see Section 3). P2X4 subunits form functional homotrimers and heterotrimers with P2X1 and P2X6 subunits in expression systems for recombinant receptors. *hP2RX4* and *mP2rx4* are alternatively spliced, but the shorter forms do not form functional channels (Kaczmarek-Hajek, Lörinczi, Hausmann, & Nicke, 2012). In addition, there are four non-synonymous coding SNPs in the *hP2RX4* gene, but only Tyr315Cys affects the receptor function (Stokes et al., 2011).

### 9.2 | Homomeric and heteromeric P2X4 receptor proteins

Native and recombinant homomeric P2X4 receptors activate rapidly and desensitize incompletely at moderate rates, both in an ATP concentration-dependent manner, and deactivate rapidly and independently of ATP concentration. Thus, the receptor functions as a non-selective cationic channel, and its permeability for Ca^2+^ is the highest among the family (Egan & Khakh, 2004). The P2X4 receptor is one of the most sensitive receptors to ATP, whereas the 315Cys-P2X4 mutant is less sensitive to ATP (Stokes et al., 2011). The homotrimeric and heterotrimeric P2X4 receptors, but not most other P2X receptors, are sensitive to ivermectin, which acts as a PAM of these channels (Zemkova et al., 2015).

Homotrimeric and heterotrimeric P2X4 receptors also undergo rapid constitutive and agonist-induced internalization into early endosomes and lysosomes and subsequent reinsertion into the plasma membrane (Bobanovic, Royle, & Murrell-Lagnado, 2002). Internalization of P2X4 receptors is clathrin- and dynamin-dependent and determined by the C-terminal interacting with adapter protein 2 (AP2); mutation of either the endocytic motif or the Tyr binding pocket of AP2 leads to accumulation of functional receptors in the plasma membrane. Native P2X4 receptors in cultured rat microglia, macrophages, and vascular endothelial cells are localized predominantly in lysosomes, where they retain their functionality and subsequently move out to the plasma membrane. This finding led to speculation about their intracellular functions (Kaczmarek-Hajek, Lörinczi, Hausmann, & Nicke, 2012).

### 9.3 | Distribution and function

P2X4 receptors are abundantly expressed in neurons and glial cells of several brain regions, including olfactory bulb, cerebral cortex, subcortical telencephalon, cerebellum, hypothalamus, thalamus, midbrain, hindbrain, and ventricular structures. These receptors are also present in spinal cord microglia, peripheral neurons, including somatosensory cortical, nodose ganglion, trigeminal, vestibular ganglion, and spinal cord neurons, in addition to Schwann cells. In the mammalian retina, the receptor was identified in both neurons and glia (Montilla, Mata, Matute, & Domercq, 2020). The neuroendocrine cells of the hypothalamus and pituitary gland and the endocrine cells of the thyroid and adrenal glands also express P2X4 receptors (Bjelobaba, Janjic, & Stojilkovic, 2015). In the cardiovascular system, they are located in cardiac and vascular smooth muscle cells and endothelial cells (Ralevic, 2015).

Numerous studies have shown a role for P2X4 receptors in allodynia associated with chronic neuropathic pain, a persistent pain arising from changes in spinal cord processing pathways (Inoue, 2019). After nerve injury, overexpression of P2X4 receptors in Schwann cells was reported to promote motor and sensory functional recovery and remyelination via brain-derived neurotrophic factor (BDNF) secretion. Furthermore, the P2X4 receptor has a role in neuroinflammation, the complex biochemical, and cellular response occurring during infections of the brain and the spinal cord, with the participation of microglia, astrocytes, and endothelial cells. Spinal cord injury, brain ischaemia, and trauma increase P2X4 receptor expression in microglial cells, which could be involved in inflamed lesions in the brain that persist for days/weeks after an ischaemic stroke. The P2X4 receptor may also play a role in neurodegenerative diseases, such as Parkinson’s disease, Alzheimer’s disease (AD) and multiple sclerosis, as they are associated with neuroinflammation that is accompanied by P2X4 receptor-dependent microglia activation. Expression of P2X4 receptors is up-regulated in activated microglia from rats with experimental autoimmune encephalomyelitis (a model of amyotrophic lateral sclerosis) and in human multiple sclerosis optic nerve samples, and they appear to facilitate repair response after demyelination (Montilla, Mata, Matute, & Domercq, 2020). Finally, activated P2X4 receptors stimulate electrical activity, Ca^2+^ signalling, and neurohormone secretion in neuroendocrine cells (Bjelobaba, Janjic, & Stojilkovic, 2015).

### 9.4 | Transgenic and KO models (see also Section 4)

A transgenic mouse model overexpressing the human P2X4 subunit exhibited increased contractility of cardiomyocytes and greater global contraction performance in intact heart compared with wild-type (WT) animals (Hu et al., 2001). Overexpression of human P2X4 receptors in a calsequestrin transgenic mouse model of cardiomyopathy significantly delayed heart failure progression and increased life expectancy by more than twofold (Yang, Sonin, Jones, Barry, & Liang, 2004). The P2X4 receptor also contributes to the control of large vessel tone through endothelial-dependent NO release and arterial smooth muscle relaxation. Consistently, P2X4 receptor KO mice and human carriers of low functional 315Cys-P2X4 receptors have raised BP (Braganca & Correia-de-Sa, 2020).

## 10 | P2X5 RECEPTOR

### 10.1 | P2RX5 gene, splice variants, and SNPs

The human *P2RX5* gene (ENSG00000083454), located on 17p13.2, occurs as two alleles: T allele and G allele. The T allele leads to mature transcription of a full-length P2X5 subunit of 444 amino acids and assembly of functional P2X5 receptor ion channels (Kotnis et al., 2010). The G allele appears in samples of human genomic DNA, but not in other mammals (Kotnis et al., 2010). The 3′-splice site (GGTCGT***gg***gat) of exon 10 contains ***gg*** on the intronic side rather than ***gt***, at which RNA splicing occurs (Bo et al., 2003). This SNP results in a shorter subunit, which lacks the 22 amino acids encoded by exon 10 (Duckwitz, Hausmann, Aschrafi, & Schmalzing, 2006; Le, Paquet, Nouel, Babinski, & Seguela, 1997). Assemblies of the exon 10-deleted hP2X5 receptor are retained in the cytosol by the endoplasmic reticulum (Duckwitz, Hausmann, Aschrafi, & Schmalzing, 2006).

### 10.2 | P2X5 receptor protein

rP2X5 and mP2X5 subunits share 95% homology over their 455 amino acid length, and 62% homology with the full-length hP2X5 subunit. rP2X5 and mP2X5 receptor channels are remarkable for producing very small currents to supramaximal ATP concentrations, around 5% of the amplitude of inwards currents generated under equivalent conditions by other P2X receptors expressed in cell lines or *X. laevis* oocytes (Collo et al., 1996). Light was shed on the cause of the small ATP responses, using a series of chimeric P2X5 receptors bearing rat peptide sequences replaced with human equivalents (Sun et al., 2019). Two rP2X5 receptor chimeras yielded considerably larger ATP responses: rP2X5 receptor-chimera 3, Ile^50^–Arg^114^ (72 pA·pF^−1^); rP2X5 receptor-chimera 5, Leu^171^–Lys^205^ (162 pA·pF^−1^); compared with rP2X5 receptor-WT (2 pA·pF^−1^). Single-substitution experiments subsequently revealed that any one of three mutations (Ser191Phe, Phe195His, and Val67Iso) significantly improved functionality of rP2X5 receptors by improving ATP binding at its docking site (Sun et al., 2019).

When stimulated by ATP, P2X5 receptors function as slowly desensitizing, non-selective cationic channels (*P*_Ca_/*P*_Na_ = 1.5). Unlike other P2X receptors, the P2X5 receptor is also permeable to chloride ions (*P*_Cl_/*P*_Na_ = 0.5) (Bo et al., 2003).

### 10.3 | P2X5 receptor function

Functional P2X5 receptors may play a supporting role in the inflammatory response. Gene deletion of P2X5 receptors (*mP2RX5*^−/−^) decreased inflammatory bone loss in the parietal calvarium (skull), in vivo, without affecting normal bone development and homeostasis (Kim et al., 2017). Additionally, expression levels of pro-inflammatory IL-1β, IL-6, IL-17a, and TNF-sf11 were significantly lower in *P2rx5*^−/−^ mice compared with WT mice (Kim et al., 2018).

## 11 | P2X6 RECEPTORS

### 11.1 | *P2RX6* gene and P2X6 receptor protein

The encoding DNA for the protein subunit has been identified in the human genome (ENSG00000099957) at 22q11.21. P2X6 receptors are ATP-gated ion channels when fully glycosylated. In 25 years, only two groups have succeeded in characterizing functional rP2X6 receptors (Collo et al., 1996; Jones et al., 2004). In both studies, around 5% of transfected HEK293 cells yielded agonist responses to ATP and other nucleotides, and P2X6 receptors functioned as slowly desensitizing, non-selective cationic channels.

*N*-linked glycosylation of ion channels can affect subunit folding, oligomeric assembly, trafficking to the membrane, agonist binding, and channel opening. The extracellular domain of P2X6 receptors contains the N*X*S/T glycosylation motif at three sites, with asparagine (N) residues at positions 157, 187, and 202 of the rat isoform (Jones et al., 2004; Newbolt et al., 1998; Rettinger, Aschrafi, & Schmalzing, 2000). Functional and non-functional rP2X6 receptor proteins extracted from HEK293 cells were discriminated by the molecular mass of epitope-tagged P2X6 subunits of 70- and 60-kDa MW, respectively (Jones et al., 2004). Treatment with *N*-glycosidase F reduced the molecular masses to 50 kDa, which is the expected size of the non-glycosylated P2X6 protein subunits. Thus, the efficiency of subunit glycosylation may hold the key to whether or not P2X6 receptors express functionally.

### 11.2 | P2X6 receptor distribution and function

There has been renewed interest in P2X6R expression of P2X6 receptors, based on two observations. Firstly, glycosylation of P2X6 subunits may be more efficient in native cell types compared with expression systems, producing the 70-kDa P2X6 subunits identified in adult midbrain, atrium, kidney, thymocytes, and urinary bladder (Jones et al., 2004). Secondly, non-glycosylated P2X6 receptors were translocated through the nuclear pore complex to the nucleus of mouse hippocampus neurons, where they interacted with the splicing factor (SF3A1), to reduce the incidence of mRNA splicing (Diaz-Hernandez, Sebastian-Serrano, Gomez-Villafuertes, Diaz-Hernandez, & Miras-Portugal, 2015). A recent clinical study has implicated P2X6 receptor overexpression in the progression and poor prognosis of renal cell cancer in human patients (Gong et al., 2019).

## 12 | P2X7 RECEPTORS

### 12.1 | The *P2RX7* gene, splice variants, and SNPs

The human P2X7 receptor is encoded by the *hP2RX7* gene (ENSG00000089041) on the long arm of chromosome 12, at 12q24.31 (Bartlett, Stokes, & Sluyter, 2014), close to the *hP2RX4* gene (12.q24.32), while *mP2rx7* is located on chromosome 5. Several non-synonymous, intronic, or missense SNPs have been reported in the *hP2RX7* gene. A number of P2X7 receptor isoforms derived from alternative splicing were identified both in humans and in rodents (Bartlett, Stokes, & Sluyter, 2014). Some variants are expressed and functional, for example, human P2X7B receptor, and mouse and rat P2X7 receptor variant “k.”

Some SNPs occurring in the coding region cause gain or loss of receptor function and are variably associated to different disease conditions. Linkage studies suggested that the SNP *rs2230912* coding for Gln460Arg-P2X7R is connected with major depression and bipolar disorder, although this has been questioned by others (Illes, Rubini, Huang, & Tang, 2019).

### 12.2 | The P2X7 receptor protein

The P2X7 receptor has the lowest affinity for ATP among all P2 receptors, a feature that has often raised doubts on its pathophysiological role. However, it is now clear that at sites of inflammation or in cancer, the local extracellular ATP concentration can rise to levels close to those needed to stimulate the P2X7 receptors (Di Virgilio, Schmalzing, & Markwardt, 2018). In addition, some inflammatory factors may act as PAMs, thus lowering the ATP threshold for P2X7 receptor activation (Di Virgilio, Sarti, Falzoni, De Marchi, & Adinolfi, 2018).

It was suggested about 20 years ago that some P2X receptor channels (P2X2, P2X4, and P2X7) exhibit progressive dilation during long-lasting stimulation by ATP and that the generated pore is permeable to high MW cationic dyes, such as NMDG, Yo-Pro, ethidium, or propidium iodide (Di Virgilio, Sarti, Falzoni, De Marchi, & Adinolfi, 2018). However, later, it was shown that this interpretation of the experimental data obtained by reversal potential measurements is probably misleading. Participation of associated channel-forming proteins, such as pannexin-1 or connexin-43, has been suggested but convincing evidence now supports the view that the P2X7 receptor itself has the ability to form a large-conductance pore in the absence of any significant dilatation. Simply, the P2X7 receptor channel allows the passage of large cationic molecules immediately from its initial activation, but at a much slower pace than that of the small cations Na^+^, K^+^, and Ca^2+^ (Di Virgilio, Sarti, Falzoni, De Marchi, & Adinolfi, 2018).

An early argument brought up to support the assumed dilation of P2X7 receptors was the facilitation of P2X7 receptor currents during long-lasting or repetitive application of ATP or BzATP (Surprenant, Rassendren, Kawashima, North, & Buell, 1996). However, this was later shown to be independent of the entry of cationic molecules via the receptor channel and rather caused by a Ca^2+^/calmodulin-dependent current facilitation through some P2X7 receptor orthologues (rat, but not human) (Roger, Gillet, Baroja-Mazo, Surprenant, & Pelegrin, 2010) or the secondary activation of a chloride current, for example, in macrophages (Janks, Sprague, & Egan, 2019).

### 12.3 | P2X7 receptors in the immune system

The P2X7 receptor is widely expressed by myeloid and lymphoid immune cells, as well as by mast cells (Di Virgilio, Dal Ben, Sarti, Giuliani, & Falzoni, 2017). Platelets also express P2X7 receptors, albeit at low level. The best characterized immune response associated with P2X7 receptor stimulation is activation of the leucine-rich repeat, pyrin domain-containing 3 (NLRP3) inflammasome, and IL-1β secretion, but several other key immune responses (e.g., release of additional pro-inflammatory or anti-inflammatory cytokines or chemokines, generation of reactive oxygen species (ROS), promotion of chemotaxis, stimulation/inhibition of phagocytosis, destruction of intracellular pathogens, and formation of multinucleated giant cell at inflammatory granulomas) are promoted by P2X7 receptor stimulation. Due to the relevance of the P2X7 receptor in macrophage responses, the association of major infectious diseases with *P2RX7* polymorphisms has been widely investigated but with inconsistent results.

The P2X7 receptor has a special place in the overall mechanism of IL-1β secretion since its gating by extracellular ATP allows the efflux of large amounts of cytosolic K^+^ that in turn drives NLRP3 assembly and caspase-1 activation. In fact, the P2X7 receptor is the most potent plasma membrane receptor triggering pro-IL-1β processing and release (Di Virgilio, Dal Ben, Sarti, Giuliani, & Falzoni, 2017) and thus is a crucial initiator of inflammation. P2X7 receptor KO mice are less prone to initiate inflammation in response to a variety of stimuli. Secretion of mature IL-1β is severely reduced, and as a consequence, initiation of the cascade of inflammatory cytokines is also impaired (Solle et al., 2001).

Human neutrophils express functional P2X7 receptors coupled to NLRP3 activation (Karmakar, Katsnelson, Dubyak, & Pearlman, 2016). In these cells, P2X7 receptor activity is required for efficient clearance of *Streptococcus pneumoniae*-sustained bacterial infection (Karmakar, Katsnelson, Dubyak, & Pearlman, 2016). Dendritic cells (DCs) are the immune cell types that express the highest level of P2X7 receptors. Different responses are dependent on P2X7 receptor function in DCs, most notably antigen presentation (Mutini et al., 1999). It is highly likely that the P2X7 receptor is a key component of the DAMP-sustained stimulatory circuit whereby adjuvants potentiate antigen presentation (Di Virgilio, Dal Ben, Sarti, Giuliani, & Falzoni, 2017).

Very recently, P2X7 receptors were shown to be necessary to establish long-lived memory CD8^+^ cells and thus play a major role in immunological memory (Borges da Silva et al., 2018). Certain NK cell subtypes also express high levels of P2X7 receptors that, as for CD8^+^ T lymphocytes, have a major role in supporting energy metabolism and maintaining these lymphoid cells fit. In the gut, P2X7 receptors are expressed by T follicular helper cells (Tfh) where they participates in Tfh–B-cell communication (Perruzza et al., 2017). P2X7 receptor activity is understood to be necessary for the differentiation of IL-17-producing T lymphocytes in models of experimental arthritis (Fan et al., 2016) and for the induction of IL-23-dependent psoriatic dermatitis (Diaz-Perez et al., 2018).

The P2X7 receptor is an important link between inflammation and coagulation since stimulation of macrophage and DC P2X7 receptors drive a large microvesicle-mediated release of tissue factor (TF), the initiating agent of the extrinsic coagulation pathway (Baroni et al., 2007). The P2X7 receptor is one of the most potent triggers for the release of exosomes and plasma membrane-derived microvesicles containing a vast array of intracellular components and exposing a variety of surface markers (Sluyter, 2017). Furthermore, stimulation of P2X7 receptors promotes vascular endothelial growth factor (VEGF) release and supports angiogenic activity in vivo (Adinolfi et al., 2012). Participation of the P2X7 receptors in cancer growth and metastasis is increasingly recognized (Di Virgilio, Schmalzing, & Markwardt, 2018).

### 12.4 | P2X7 receptors in peripheral organs

Outside the immune system, the P2X7 receptor is expressed by many different cell types, such as keratinocytes, corneal cells, hepatocytes, intestinal epithelial cells, vascular endothelial cells, retinal ganglion cells, fibroblasts, osteoclasts, osteoblasts, vascular smooth muscle, and skeletal muscle (Bartlett, Stokes, & Sluyter, 2014; Sluyter, 2017). It can be safely concluded that P2X7 receptors are ubiquitously expressed throughout the body, albeit to different levels. While the role of the P2X7 receptors in the immune system is well established, in other tissues, it is more elusive.

### 12.5 | P2X7 receptors in the CNS

The mammalian CNS consists of neuronal and non-neuronal cells. The latter comprise mainly of glia (astrocyte-like cells, oligodendrocytes, and microglia) and ependymal cells. Microglia are resident macrophages of the CNS, similar in function to blood-born peripheral immunocytes (monocytes/macrophages and lymphocytes), which cross the blood–brain barrier (BBB) only in case of massive infections or BBB damage of diverse origin. Microglia possess the highest density of P2X7 receptors, surmounting that present in astrocytes/oligodendrocytes, whereas neurons appear to be devoid of this receptor (Illes, Khan, & Rubini, 2017). Effects attributed previously to the activation of neuronal P2X7 receptors are now thought to be indirect, mediated by the release/outflow of gliotransmitters, or other types of glial signalling molecules (Illes, Verkhratsky, & Tang, 2019).

### 12.6 | Involvement of P2X7 receptors in neurological and psychiatric illnesses

Although neurodegenerative illnesses have specific and distinct causative factors, they generate also an additional neuroinflammatory component that aggravates the primary condition. P2X7 receptors of glial cells are intimately involved in neuroinflammation, and therefore, P2X7R antagonists have a favourable symptomatic impact in case of these illnesses. Glial (especially microglial) P2X7Rs are stimulated by large concentrations of ATP released from CNS cells under noxious conditions, during both acute injury (trauma, hypoxia/ischaemia, and epilepsy-induced seizures) and chronic neurodegenerative conditions (AD, Parkinson’s disease, amyotrophic lateral sclerosis, and multiple sclerosis) (Burnstock, Krügel, Abbracchio, & Illes, 2011).

Epilepsy is typically thought to be the result of an enduring imbalance between excitation and inhibition in the brain (Engel, Alves, Sheedy, & Henshall, 2016). A major cause of long-lasting epileptic seizures termed “status epilepticus” (SE) is caused by the increased release of ATP. Kainic acid injection into the nucleus amygdala of mice induced seizure-like EEG activity; P2X7 receptor antagonists reduced electrographic seizures and cortical cell death. Conversely, when SE was induced by the systemic injection of pilocarpine, immediate seizure activity was increased, recurrent seizures following a one-time pilocarpine injection were facilitated, and this was thought to be due to the enhanced survival of hippocampal neural progenitor cells migrating into ectopic locations and generating a pathological pacemaker (Rozmer et al., 2017).

The production of the neurotoxic molecules, β-amyloid (Aβ), and hyperphosphorylated tau have been assumed to cause AD and the cardinal clinical symptom, cognitive deterioration (Illes, Burnstock, & Tang, 2019). A diversity of investigations with primary microglial cultures and in vivo AD animal models suggested that Aβ induces an increase in [Ca^2+^]_i_ in microglia via P2X7 receptor stimulation and the subsequent massive release of ATP (Sanz et al., 2009). A possible chain of events is that ATP released from damaged CNS cell types stimulates microglial P2X7 receptors and releases IL-1β, as well as other cell products. The beneficial effects of BBB-permeable P2X7 receptor antagonists in AD animal models also support the notion of pathological microglial activation in AD (Csölle et al., 2013).

As already mentioned, the SNP Gln460Arg-P2X7 receptor is thought to be a predisposing factor for the affective diseases, major depression and bipolar disease. In addition, various models of inescapable rodent stress lead to the generation of a behavioural reaction termed “learned helplessness” (Illes, Rubini, Huang, & Tang, 2019). This is believed to be due to the increased secretion of adrenocorticotropic hormone (ACTH), and subsequently glucocorticoids, thought to cause depressive-like behaviour. Co-stimulation of microglial toll-like receptors (TLRs) and P2X7 receptors by DAMPs (ATP itself is the most ubiquitous DAMP) triggers NLRP3 activation and the associated IL-1β release, which is probably a major stimulus for the corticotropin-releasing hormone-induced activation of the hypothalamic–pituitary–adrenal axis. In fact, P2X7 receptor KO mice by themselves and WT mice after the application of P2X7 receptor antagonists exhibited an anti-depressant-like profile in animal models of major depression and bipolar disorder (Basso et al., 2009).

## 13 | CONCLUSIONS

P2X receptors appear very early in phylogeny (algae, amoeba, and basal fungi), and it is a fascinating property of these primitive receptors that, in contrast to their mammalian counterparts, they are expressed predominantly or even exclusively intracellularly. The mammalian P2X1–7 receptors reside mainly in the plasma membrane, and their ATP-induced opening and the subsequent cationic fluxes can regulate many essential cellular functions. Our knowledge of P2X receptor structure/function has greatly increased in the four decades elapsing since their discovery. Crystallization of the truncated zfP2X4 receptor disclosed the structure of a chalice-shaped trimeric receptor that allows cations to flow through fenestrations to the vestibules near the ion channel, resulting in TM ion fluxes. The zfP2X4 receptor served as a pattern for homology modelling of mammalian P2X receptor subtypes before they were crystallized and their 3D structure resolved.

Medicinal chemistry has made major contributions to the field by synthesizing subtype-selective antagonists and, with some temporal delay, PAMs and NAMs. This is a prerequisite of any conclusive experimental work and was therefore essential for moving the field ahead. Some of these antagonists were already prospective drugs in that they had favourable bioavailability after oral application and in case of an intended CNS activity, avidly crossed the BBB. Indispensable for research activities was the generation of a battery of transgenic animals. Reporter mice that express soluble fluorescent reporter proteins or fluorescent protein-tagged P2X receptors allowed targeted investigations of receptor-containing cells or their subcellular compartments.

Especially important was the assignment of deficiencies in individual P2X receptor subtypes to certain diseases with a genetic background and more recently population genetic studies aimed at the identification of loss-of-function SNPs pathogenically involved, for example, in affective diseases. In conclusion, structural deficiencies of P2X receptors may underlie certain illnesses, and on the contrary, selective antagonists or NAMs may correct the deleterious consequences of a pathological overstimulation by ATP (e.g., neuropathic/inflammatory pain or neurodegenerative illnesses). Although a P2X receptor-based, widely used, drug is still missing in our therapeutic repertoire, the pharmaceutical industry is working intensively in this field and there is strong hope of achieving a major breakthrough in the near future (Cully, 2020; Krajewski, 2020).

### 13.1 | Nomenclature of targets and ligands

Key protein targets and ligands in this article are hyperlinked to corresponding entries in the IUPHAR/BPS Guide to PHARMACOLOGY (http://www.guidetopharmacology.org) and are permanently archived in the Concise Guide to PHARMACOLOGY 2019/20 (Alexander, Christopoulos et al., 2019; Alexander, Fabbro, et al., 2019; Alexander, Mathie et al., 2019).

## Figures and Tables

**TABLE 1:** Properties of recombinant homomeric P2X receptors and their pharmacology

	NC-IUPHAR subunit nomenclature						
	P2X1	P2X2	P2X3	P2X4	P2X5	P2X6	P2X7
Molecular properties^a^							
Gene name	*P2RX1*	*P2RX2*	*P2RX3*	*P2RX4*	*P2RX5*	*P2RX6*	*P2RX7*
Human chromosome location	17p13.3	12q24.33	11q12.1	12q24.32	17p13.3	22q11.21	12q24.31
Protein length (amino acids)	399	471	397	388	444	441	595
C tail length (amino acids)	41	113	56	29	82	87	240
Membrane expression	Good	Good	Good	Good	Poor	Usually no expression	Good
Desensitization (complete in)	Fast (<1 s)	Slow (>20 s)	Fast (<1 s)	Slow (>20 s)	Slow (>20 s)	—	Slow (>20 s)
Activation-dependent endocytosis	Yes	No	—	Yes	—	—	—
Pharmacology^a^							
Agonists (EC_50_ values [μM]), Ref. 1, Ref. 7							
ATP **1**	0.56–0.70	2–8	0.5–1	1–10	0.44–10	12	100 (r)
2-MeSATP **2**	0.07–1	1	0.35	0.29; 4.5 (r);	0.5–10	9	178;
				1.35 (m)			2,000–4,000 (m)
ATPγS **3**	2.3	1.5 (r)	0.7	2.3 (r)	0.5; 0.6 (r)	1.3 (r)	138
α,β-MeATP **4**	0.1–1	>300	0.74; 1–2 (r)	0.81; >100 (r, m)	160 to >300; 1.1 (r)	>100	>300
β,γ-MeATP **5**	2	>300	>300 (r)	>300	11.8 (r)	—	—
BzATP **6**	0.002	0.75	0.08	0.5; >100 (r); 2.9 (m)	6–40; >500;	25	5; 10–52 (r)
					(partial, r)		
Antagonists (IC_50_ values [μM])							
Non-selective antagonists							
Suramin, Ref. 1	1–2	10	3	>300	2–3	—	>300
PPADS	1	1	1	>500	3	>100	10–50
RB-2 **14b**, Ref. 1	30 (r)	0.4–0.5	-		18.3 (r)	—	—
Aurintricarboxylic acid	0.0086 (r)	21.7 (r)	0.0729 (r)	763 (r)	—	—	118 (r)
Nucleotide-derived antagonists							
TNP-ATP **7**, Ref. 1, Ref. 5	0.006	1–2	0.001	1.5–15	0.45 (r)	—	>30
2’,3’−0-Benzylidene-ATP **8**	0.002	5.5	0.08	0.49	40	25	52
DT-0111 **9**	—	—	0.3 (P2X2/3)	—	—	—	—
IP5I **10**, Ref. 1	0.003	>300	2.8	Potentiation	>30 (r)	—	
Non-nucleotide-derived antagonists							
P2X1 receptor antagonists							
PSB-2001 **16a**	0.019	>10	>10	0.156	—	—	0.175
PSB-2014 **16b**	0.0231	>10	>10	0.209	—	—	0.196
PSB-2013 **16c**	0.058	>10	>10	0.049	—	—	0.177
NF279 **17**, Ref. 1	0.02–12	1	2	>300	—	—	3–20
NF023 **18**, Ref. 1	0.2	>50	28.5; 8.5 (r)	>100	—	—	—
MRS2159 **19**	80	>100	>100	—	—	—	>100
P2X2 receptor antagonists							
NF770 **20**	1	0.019	0.08	>100	—	—	>100
PSB-10211 **21**, Ref. 2	—	0.09 (r)	—	—	—	—	—
PSB-1011 **22**, Ref. 2	0.42 (r)	0.08 (r)	0.49 (r)	>10 (r)	—	—	>10(r)
P2X3 receptor antagonists							
A-317491 **23**	>10	>100	0.1	>100	—	>100	>100
Gefapixant (AF-219, MK-7264) **24**, Ref. 4	>10	0.100–0.250	0.03	>10	>10 (r)	—	>10
		(P2X2/3)					
AF-353 (RO-4) **25**	>10	>10	0.006	—	>10	—	>10
AF-906 (RO-51) **26**	>10	>10	0.002	>10	>10		>10
BLU-5937 **27**, Ref. 3, Ref. 4	>20	>24 (P2X2/3)	0.025; 0.092 (r)	>20	—	—	>20
P2X4 receptor antagonists							
5-BDBD **28**, Ref. 7	>10 (r)	>10 (r)	>10(r)	0.35–0.5; 3.5 (r);	—	—	>10(r)
				2.5 (m)	—	—	
NP-1815-PX **29**	>30	7.3	>30 (r)	0.26	—	—	>30
PSB-12054 **30**, Ref. 5	6.5	>10	>10	0.19	—	—	>10
PSB-12062 **31**, Ref. 5	>10	>10	>10	1.4	—	—	>10
BX430 **32**	>10	>10	>10	0,78	>10	—	>50
BAY-1797 **33**, Ref. 6	>50	>30/P2X2/3	8.3	0.11–0.23 (h, m, r)	—	—	10.6
PSB-15417	10.3	>10	4.14	0.022; 0.037 (r);	—	—	2.13
				0.087 (m)			
P2X7 receptor antagonists						—	
A-438079 **34**, Ref. 1	>100	>100	>100	>100	—	>100	0.06–0.5
A-804598 **35**	>100	>100	>100	>100	—	>100	0.010 (h, r, m)
A-740003 **36**, Ref. 1	>100	>100	>100	>100	—	>100	0.04–0.05; 0.02 (r)
AZ11645373 **37**	>10	>10	>10	>10	>10	—	0.1
CE-224,535 **38**	>10	—	—	—	—	—	0.002–0.013; inactive (rodent)
GSK1482160 **39**	—	—	—	—	—	—	0.003
AZ9056 **40**	—	—	—	—	—	—	0.012
AZ10606120 **41**	—	—	—	—	—	—	0.0014
JNJ47965567 **42**	—	—	—	—	—	—	0.005
JNJ54175446 **43**	—	—	—	—	—	—	0.003
[^11^C]JNJ-54173717 **44**	—	—	—	—	—	—	0.0016
[^18^F]JNJ-64413739 **45**	—	—	—	—	—	—	0.015
Positive allosteric modulators (EC_50_ values [μM])							
Ivermectin **11**	—	>30	>30	0.25	—	—	>30 (r, m), h↑
MRS2219 **12**	5.9 (r)	>100 (r)	>100 (r)	>100 (r)	—	—	—
PSB-10129 **13**	-	0.489	—	—	—	—	—
Cibacron Blue **14a**	IC_50_ = 0.7 mM	—	Potentiation	Potentiation, block	—	—	—
Ginsenoside C-K **15**	—	—	—	8.5	—	—	1.1
Modulatory cations							
Zn^2+^	—	Increase EC_50_ = 7 μM	-	Increase 2 μM	—	—	—
H^+^	Decrease pKa 6.3	Increase pKa 7.3	Decrease pKa 6.0	Decrease pKa 6.8	—	—	Decrease pKa 6.1
Physiology and pathophysiology							
Major cellular expression	Smooth muscle	Neurons	Pain-sensing neurons	Neurons, microglia	Skeletal muscle	Broad expression	Immune cells
Major role	Neuroeffector transmission	Taste, hearing	Pain, bladder reflexes, taste	Vascular remodelling, neuropathic pain	Inflammatory bone loss	—	Inflammation, neurodegenerative illnesses
Model native cell type	Vas deferens, myocytes	SCG, myenteric plexus neurons	Small DRG neurons	Macrophages	Skeletal myocytes	—	Monocytes, macrophages, microglia
KO available?	Yes	Yes	Yes	Yes	Yes	Yes	Yes

*Note*: The pharmacological values apply to homotrimeric receptors, unless noted. Bold numbers refer to chemical structures shown in Figures 1 and 2. Modified from Table 1 of Jarvis and Khakh (2009) with permission. Additional references are marked with numbers as follows: Ref. 1, Lambertucci et al. (2015); Ref. 2, Baqi et al. (2011); Ref. 3, Garceau and Chauret (2019); Ref. 4, Marucci et al. (2019); Ref. 5, Hernandez-Olmos et al. (2012); and Ref. 6, Werner et al. (2019).

Abbreviations: DRG, dorsal root ganglion; KO, knockout; NC-IUPHAR, International Union of Basic and Clinical Pharmacology Committee on Receptor Nomenclature and Drug Classification; SCG, superior cervical ganglion.

a Human (h) P2X receptor, unless noted (m, mouse; r, rat).

## Abbreviations:

AD: Alzheimer’s disease
BBB: blood–brain barrier
BzATP: dibenzoyl-ATP
DRG: dorsal root ganglion
h: human
KO: knockout
m: mouse
NANC: non-adrenergic, non-cholinergic
PAM: positive allosteric modulator
r: rat
SE: status epilepticus
SNP: single nucleotide polymorphism
TM: transmembrane
TNP-ATP: trinitrophenyl-ATP
α,β-meATP: α,β-methylene ATP
